# Cosine similarity knowledge distillation for surface anomaly detection

**DOI:** 10.1038/s41598-024-58409-9

**Published:** 2024-04-08

**Authors:** Siyu Sheng, Junfeng Jing, Zhen Wang, Huanhuan Zhang

**Affiliations:** 1 College of Electrical and Information, Xi’an Polytechnic University, Xi’an, 710048 China; 2 Xi’an Polytechnic University Branch of Shaanxi Artificial Intelligence Joint Laboratory, Xi’an, 710048 China; 3 Defense Innovation Institute, Beijing, 100071 China

**Keywords:** Mechanical engineering, Computer science

## Abstract

The current state-of-the-art anomaly detection methods based on knowledge distillation (KD) typically depend on smaller student networks or reverse distillation to address vanishing representations discrepancy on anomalies. These methods often struggle to achieve precise detection when dealing with complex texture backgrounds containing anomalies due to the similarity between anomalous and non-anomalous regions. Therefore, we propose a new paradigm—Cosine Similarity Knowledge Distillation (CSKD), for surface anomaly detection and localization. We focus on the superior performance of the same deeper teacher and student encoders by the distillation loss in traditional knowledge distillation-based methods. Essentially, we introduce the Attention One-Class Embedding (AOCE) in the student network to enhance learning capabilities and reduce the effect of the teacher–student (T–S) model on response similarity in anomalous regions. Furthermore, we find the optimal models by different classes’ hard-coded epochs, and an adaptive optimal model selection method is designed. Extensive experiments on the MVTec dataset with 99.2% image-level AUROC and 98.2%/94.7% pixel-level AUROC/PRO demonstrate that our method outperforms existing unsupervised anomaly detection algorithms. Additional experiments on DAGM dataset, and one-class anomaly detection benchmarks further show the superiority of the proposed method.

## Introduction

In industrial production processes, surface defect detection is typically defined as the task of finding and ideally localizing anomalies in images that closely align with the training, i.e., differ only in minute deviations potentially confined to small, isolated areas. These defective images that differ from normal images are also considered anomalies, which makes surface defect detection also called anomaly detection. Surface anomaly/defect detection on the images collected by these industrial products, including the detection and location of defects in these industrial images (that is, measurement, including defect position, size and other information), has become an important role of quality inspection. Surface anomaly detection techniques find broad application in diverse image-centric domains, including industrial product quality control and health management^1–4^. In industrial production, the number of abnormal images and the difficulty of manual annotation are limitations of existing supervised surface anomaly detection methods. Therefore, current research focuses on unsupervised surface anomaly detection methods (such as knowledge distillation-based^5–7^, feature matching^8–11^, and image reconstruction methods^12–16^) that only require non-anomaly samples for training.

**Figure 1:**
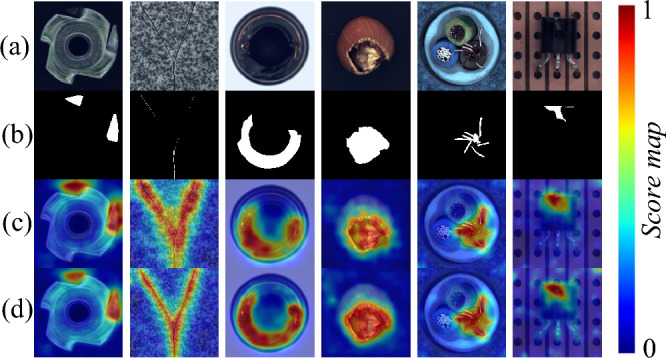
Surface anomaly detection results of challenging samples in MVTec AD^17^. Rows (**a**) and (**b**) are the input image and ground truth, respectively. Rows (**c**) and (**d**) are the anomaly scores of RD^18^ and ours.

In this study, we concentrate on the unsupervised surface anomaly detection problem and approach it through the lens of traditional knowledge distillation techniques. Knowledge distillation-based^6,7^ methods are based on the assumption that teacher and student networks’ discrepant representations of input samples to achieve anomaly detection and localization. Currently, most of the studies are focused on using smaller student in multiresolution knowledge distillation (MKD)^5^ or reverse distillation (RD)^18^ with encoder–decoder architecture to solve the problem of vanishing representations discrepancy on anomalies (i.e. boosting the diversity of anomalous representations^18^). Nonetheless, these strategies are accompanied by several shortcomings. Primarily, the smaller student models struggle to completely extract both low-level structural and high-level semantic representations from the input. Furthermore, reverse distillation encounters difficulties in accurately reconstructing anomaly-free regions in complex texture backgrounds, as the student network fails to utilize the low- and high-level representations derived from the teacher effectively. These methodological constraints ultimately impede the overall performance of the model.

To comprehensively address the above problems, we introduce a novel framework based on traditional knowledge distillation, *Cosine Similarity Knowledge Distillation*. (1) we regress the encoder–encoder with the same depth, then use the differential representations generated by the hypothetical pre-trained teacher network and the unpre-trained student under the new distillation loss. (2) The proposed Attention One-Class Embedding (AOCE) module composed of the assistant student (AS) and one-class embedding (OCE) block which are applied in the student network. The AS facilitates the student’s imitation of the teacher’s behavior. During the querying/testing, for the abnormal representations extracted by the student, OCE is used to smooth abnormal information. Furthermore, our method is no longer limited to models that obtain the same number of epochs for all classes of targets with fixed parameters (Fig. 1 shows the qualitative results of our method).

We perform extensive experiments with the MVTec dataset^17^, DAGM dataset^19^, and one-class novelty detection datasets. Compared with Reverse Distillation and other related unsupervised surface anomaly detection methods, the experimental results show that the proposed model with the AOCE module surpasses existing methods to a certain extent. The primary contributions of this paper can be outlined as follows:

- We present a novel *Cosine Similarity Knowledge Distillation* approach specifically designed for surface anomaly detection. The encoder–encoder architecture of the same depth is applied to increase the learning ability of the student model on feature representations. To counteract incorrect extraction of abnormal information by the student model, we propose an *AOCE* module as a distinguishing filter to prevent the vanishing representations discrepancy between teacher–student pairs, leading to improve the performance of the model.
- Assistant student and the proposed one-class embedding block to form the AOCE module for feature differentiation. The assistant student strengthens the network’s focus on relevant target areas and suppresses extraneous information, whereas the one-class embedding block efficiently sifts out useless information.
- We develop an adaptive optimal model selection strategy that chooses the optimal model variant for each object category under a more stable and dependable distillation loss. This guarantees both versatility and accuracy in anomaly detection and localization endeavors.
- Extensive experimentation on benchmark datasets for unsupervised surface anomaly detection and localization confirms that our proposed method attains state-of-the-art results..

**Figure 2:**
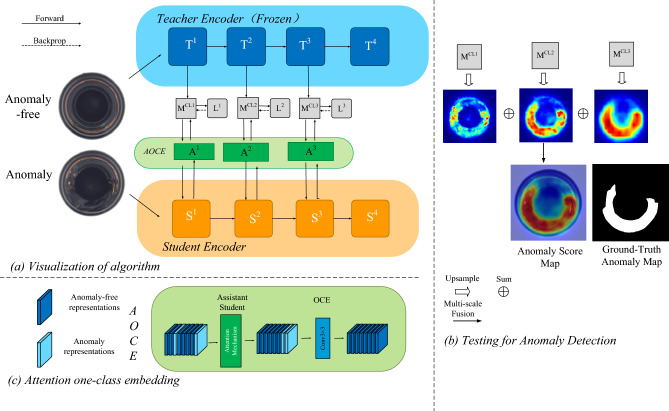
(**a**) Overview of our Cosine Similarity Knowledge Distillation framework for surface anomaly detection and localization. The proposed method uses the activation representations output by the initial three critical layers of the same teacher and student network (Wide-ResNet-50^20^). During the training phase, the student S embedded with the AOCE module imitates the teacher T from the direction by minimizing the cosine similarity loss L. (**b**) during the testing/query stages, the corresponding critical layers of T and S obtained low similarity maps under the similarity loss calculation are upsampled and then fused at multi-scale to achieve accurate detection and localization of anomalies. The ultimate prediction output is determined by the cumulative effect of these multi-scale feature maps collectively referred to as M. (**c**) The AOCE module, combines an assistant student submodule with the OCE (One-Class Embedding) block, functioning synergistically to enhance the student network’s learning aptitude and its ability to discriminate between essential and non-essential information.

## Related work

Unsupervised learning methods for surface anomaly detection and localization are generally classified into two categories, reconstruction-based methods and feature-based methods.

*Reconstruction-based methods* fundamentally depend on the differences between the input image and its reconstructed version to localize anomalies. Notable examples include Auto-Encoders (AE)^13,21–23^, which are extensively employed due to their ability to recreate the original image. Similarly, Generative Adversarial Networks (GANs)^14–16,24,25^ are commonly utilized in this context. However, the very nature of deep neural networks capable of accurately reconstructing normal images often inadvertently leads to plausible reconstructions of anomalous regions as well, thereby limiting the detection accuracy of these methodologies. DRÆM^26^ has been proposed to alleviate the issue of overfitting to synthetic anomalous patterns by training dual sub-networks—one for image reconstruction and another for discrimination. However, the precise inpainting of anomalous images makes them computationally expensive, and the randomness of the synthetic appearance also makes the performance of the models vary widely under the same training conditions. Our prior work, MÆIDM^12^, aimed to build upon DRÆM to enhance detection accuracy, yet computational efficiency remains a challenge. Different from the conventional encoder–encoder architecture, Reverse Distillation^18^ applies the idea of reconstruction to the architecture of knowledge distillation. RD’s student decoder uses low- and high-level feature representations to reconstruct non-anomalous representations in the feature subspace to achieve the difference between the teacher encoder and the student decoder. DeSTSeg^27^, which integrates a pre-trained teacher network, a denoising student encoder–decoder, and a segmentation network within a unified framework. This approach introduces a denoising procedure to enhance the robustness of the student network’s representations and adaptively fuses multi-level T–S features through rich supervision from synthetic anomaly masks.

*Feature-based methods* employs pre-trained deep learning models to derive discriminative features from either the entire image^28^ or specific image patches^8–11^ for the purposes of anomaly detection and localization. Given the paucity and unpredictable nature of anomalies, classical anomaly detection algorithms^29–31^ typically cast the problem as a one-class classification task, relying solely on normal samples for training. Deep SVDD^28^ and Patch SVDD^31^ deploys the neural network to process high-dimensional image data. On the other hand, methods like SAPDE^8,9^ and PatchCore^10^ use the non-anomalous embedding vectors obtained by feature extraction in the training set to construct a feature pool. PaDim^11^ calculates the Mahalanobis distance to gauge the dissimilarity between anomalies and their corresponding normal patch embeddings. Nevertheless, the computational complexity of these techniques generally scales linearly with the size of the training dataset. CutPaste^32^ as a two-stage framework applies data augmentation to feature-based methods to build an anomaly detector. A pre-trained deep neural network is incorporated to extract feature representation data of non-anomaly images and synthetic unreal anomaly images to train a one-class classifier to better face real-world anomalies during testing. Models fail to detect and locate large defects or structural anomalies due to limitations of synthetic appearance. A A recent development, SimpleNet^33^ is proposed for anomaly detection and localization. By integrating a pre-trained feature extractor, feature adapter, synthetic anomaly generation, and a basic binary discriminator, SimpleNet surpasses earlier methods, achieving best performance on anomaly detection tasks while maintaining a high processing speed. This addresses some of the limitations of the previous approaches, particularly regarding the handling of larger and more complex anomalies.

Another feature-based unsupervised anomaly detection approach is knowledge distillation^5,18,34–36^. Reverse distillation^18^ uses the encoder–decoder to solve the problem that the same data flow in the T–S model. RD++^34^ combines RD with multi-task learning to solve the task of anomaly signal suppression by simulating pseudo-anomalous samples through simplex noise and minimizing reconstruction loss.MKD^5^ uses a smaller clone network as the student to imitate the output of the teacher network. The student composed of the shallow network has a weaker representation ability for the input image, which makes the model not good for real-world anomaly detection performance. This paper uses the same deep neural network as T–S based on traditional knowledge distillation to better represent the low- and high-level information of the input. The proposed method also introduces an AOCE module in the student model to be a distinguishing filter and increase variance in T–S representations of abnormal regions.

## Proposed approach

In this section, we will give a detailed introduction to the proposed cosine similarity knowledge distillation framework. Firstly, cosine similarity knowledge distillation is introduced. Then, the proposed AOCE module is elaborated. Anomaly detection and localization of CSKD is finally introduced.

### Cosine similarity knowledge distillation

In the context of unsupervised surface anomaly detection, traditional knowledge distillation relies on the assumption of differential representations generated between teacher–student models to achieve anomaly detection and localization (Fig. 2 depicts the proposed cosine Similarity knowledge distillation framework for anomaly detection). For the same or similar teacher and student networks without distinguishing filters^5^. Previous work used smaller student networks or introduced encoder–decoder architectures to address this problem. It is noteworthy that these methods are not always effective in practical application, since (1) a smaller student network is associated with weaker representation ability and (2) in situations where abnormalities blend seamlessly with intricate textures in the background, the student network’s competence to faithfully reconstruct the low-level structural details of the input in the feature domain is often inadequate, further compromising its ability to detect such anomalies effectively.These factors contribute to the need for advanced and specialized approaches like the proposed Cosine Similarity Knowledge Distillation framework, which seeks to overcome these challenges and enhance the performance of unsupervised surface anomaly detection systems.

In order to tackle the primary issue of weak T–S pair representations within the knowledge distillation architecture, this study employs deeper networks Wide-ResNet-50^19^ pre-trained on ImageNet^37^ and not pre-trained as teacher and student, respectively. To ensure the teacher model maintains a stable and informative representation, we use a teacher with all parameters frozen during the distillation process, preventing convergence to a trivial solution.

**Figure 3:**
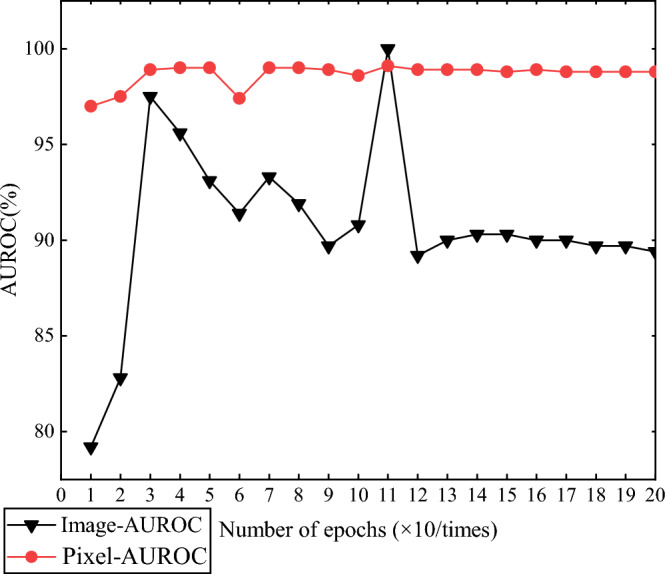
The performance of toothbrush in the MVTec^17^ dataset at different query periods, The y-axis indicates Image- and Pixel-level AUROC(%) and x-axis is the number of epochs ($\times 10/times$).

Inspired by MKD^5^ which demonstrates method stability by reporting mean and variance over the last 10 epochs for 10 distinct runs, we integrate elements from RD^18^ by introducing a query set, akin to a test set, that includes both anomalous and normal samples. This strategy improves the model’s adaptability to real-world scenarios by enhancing its anomaly detection and localization abilities during training. We combine the above work and extend it, given a batch of *n* anomaly-free images $X^t=\{X_1^t,\ldots ,X_n^t\}$ as the training set, and the same as RD^18^, we also use $X^q=\{X_1^q,\ldots ,X_n^q)\}$ as the query/test set containing both anomaly and anomaly-free images to be the disturbance. The model is trained exclusively on the anomaly-free samples from the training set, but it is evaluated against the query dataset every 10 epochs. Based on the queried evaluation metrics, we can identify the optimal hard-coded epoch with relative accuracy, thereby enhancing the stability and effectiveness of the model for anomaly detection. This is particularly important since prolonging training beyond certain epochs can degrade performance, as observed in^38^. Figure 3 illustrates the image-level and pixel-level performance of the model on toothbrush images from the MVTec dataset at various query epochs. The model peaks at the 110th epoch. Concurrent ablation experiments in section “Ablation analysis” explore the influence of different querying intervals on the chosen hard-coded epochs.

In the context of distillation loss functions, the work of MKD^5^ has shown the efficacy of incorporating cosine similarity alongside the Euclidean distance in their loss function, demonstrating the advantage of cosine similarity within the traditional knowledge distillation setup. Building upon this, RD^18^ underscores the effectiveness of using cosine similarity alone, proving that this measure effectively represents the correlation between the low-dimensional and high-dimensional representations in the reversed encoder–decoder architecture during the knowledge distillation process.

On distillation loss, MKD^5^ uses hyperparameters combined with Euclidean distance and cosine similarity method as loss function to demonstrate the superiority of the cosine similarity method under traditional knowledge distillation architecture. Building upon this, RD et al.^18,27,34^ underscores the effectiveness of using cosine similarity alone, proving that this measure effectively represents the correlation between the low-dimensional and high-dimensional representations in the reversed encoder–decoder architecture during the knowledge distillation process. Therefore, in this paper, we only use cosine similarity as the KD loss of the T–S model. Mathematically, let $CL^i$ indicate the i-th critical layer in the networks($CL^1$ stands for the first critical layer) and $x \in X^t$, the teacher encoder activation tensor of that critical layer as $A_T^{CL^i}(x)$ and the student’s as $A_S^{CL^i}(x)$. The paired feature tensor $\{A_T^{CL^i}(x), A_S^{CL^i}(x)\} \in R^{C_{CL^i} \times H_{CL^i} \times W_{CL^i}}$. $C_k$ and $H_{CL^i} \times W_{CL^i}$ denote the channel number and spatial dimension, respectively. We first calculate the cosine similarity loss of $A_T^{CL^i}(h,w)$ and $A_S^{CL^i}(h,w)$ (respectively from feature tensors $A_T^{CL^i}(x)$ and $A_S^{CL^i}(x)$ along the channel axis) to obtain 2-dimensional anomaly score maps $M^{CL^i}(h,w)$.

1 $$\begin{aligned} \begin{aligned} M^{CL^i}(h,w) = 1 - \frac{A_T^{CL^i}(h,w)^T )\cdot A_S^{CL^i}(h,w)}{\Vert A_T^{CL^i}(h,w) \Vert \Vert A_S^{CL^i}(h,w) \Vert } \end{aligned} \end{aligned}$$

where *h*, *w* represents the spatial coordinates on the feature map. When $M^{CL^i}$ assumes a substantial value, it denotes an exceptional degree of anomaly at the specific location. The total loss $L_{CS}$ guiding the student model’s optimization is the sum of distances at multi-scale feature levels.

2 $$\begin{aligned} \begin{aligned} L_{CS} = {} \sum _{i=1}^I \left( \frac{1}{H^{CL^i} W^{CL^i}} \sum _{h=1}^{H^{CL^i}} \sum _{w=1}^{W^{CL^i}} M^{CL^i}(h,w)\right) \end{aligned} \end{aligned}$$

*I* indicates the number of critical layers used in the experiment. Here are $I=3$, due to (1) deeper critical layers will lose more localized nominal information^11^ and (2) the very deep and abstract features extracted by networks pre-trained on ImageNet are biased towards natural image classification tasks^10^. Therefore, the first three critical layers of Wide-ResNet50^19^ containing low-dimensional structure and high-dimensional semantics information are selected in this paper. Ablation experiments demonstrate that this fusion outperforms both a single critical layer and other combinations of multi-scale fused critical layers.

### Attention one-class embedding

Surface anomaly detection methods rooted in knowledge distillation often encounter two main challenges. First, when the teacher and student models are built using the same deep learning architecture, the similarity of their representations in anomalous areas can lead to misdetections. Second, using a smaller student model in the teacher–student (T–S) configuration naturally compromises its representation power, which affects its ability to accurately capture normal regions. For the application of knowledge distillation tasks in anomaly detection, we hope that the student model can focus on non-abnormal regions and ignore abnormal representations, but this is not easy to achieve. Therefore, we propose the attention one-class embedding (AOCE) module as an assistant module to help students realize it. The AOCE module introduces an Assistant Student (AS) component to aid the student model in focusing on the teacher’s activation representations across the spectrum from low-dimensional to high-dimensional semantic features during both training and testing phases. This auxiliary learner helps the student extract more comprehensive and detailed representations, thereby enhancing the model’s precision in localizing anomalies. We incorporate an attention mechanism module into the AS, acting as an assistant teacher. This mechanism empowers the network to allocate more emphasis to relevant target areas while downplaying irrelevant or noisy information.

**Figure 4:**
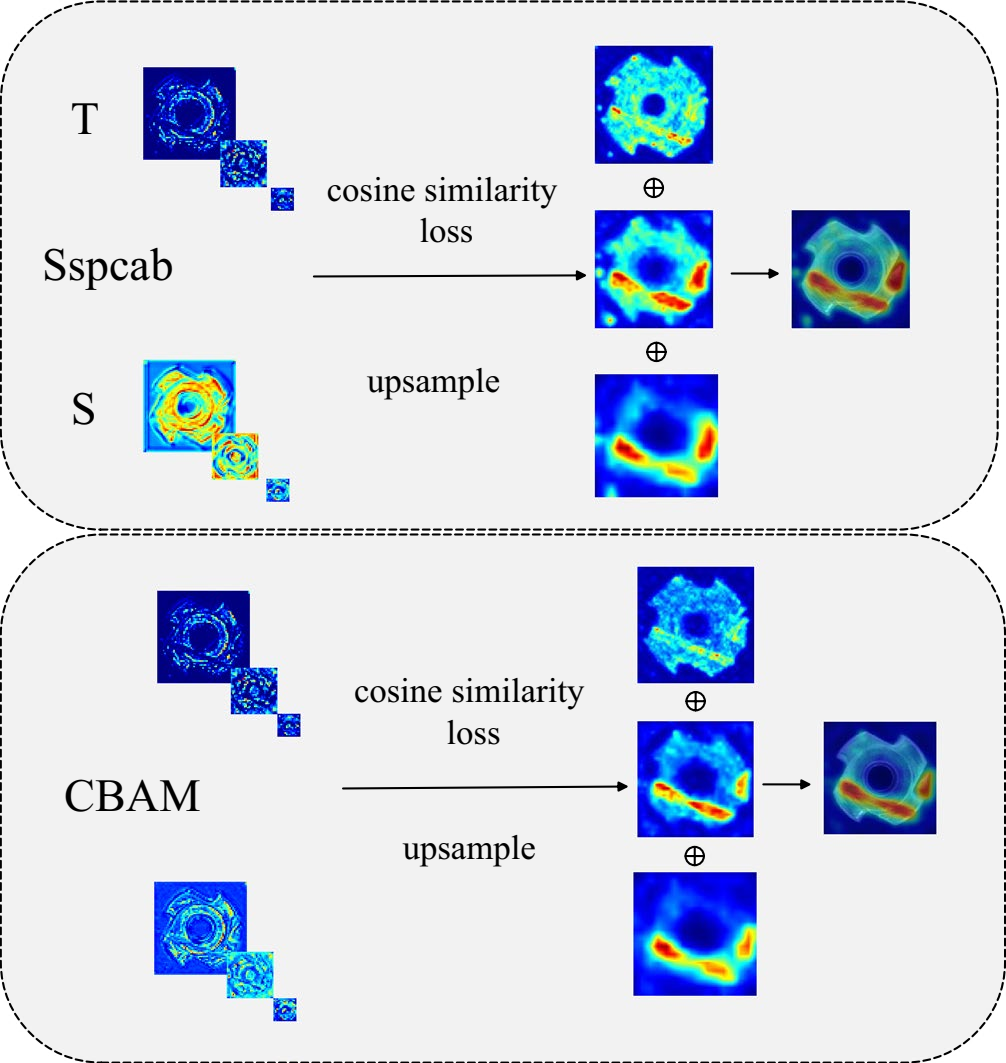
Sspcab^39^ and CBAM^41^ are used as activation representations extracted from different critical layers in AS and the results after processing by cosine similarity method.

In previous research, Sspacb^39^ was developed by integrating a channel attention module inspired by SeNet^40^ with a self-supervised reconstruction module, making it seemingly ideal for our anomaly detection needs. However, when applied to certain object categories in the MVTec dataset, such as metal nuts (as illustrated in Fig. 4), the self-supervised reconstruction module in Sspacb struggles to accurately reconstruct anomaly-free regions in the low-dimensional structural representations. This limitation negatively impacts the model’s ability to correctly detect non-anomalous samples. To address this issue, we use another attention mechanism module as AS to detect different target categories, and Fig. 4 shows the effectiveness of AS module composed of CBAM^41^ in metal nut. Meanwhile, for different target categories, SeNet^40^, and EcaNet^42^ as alternative AS modules to obtain an optimal model with relatively balanced image-level and pixel-level for anomaly detection and localization. We demonstrate the performance of different AS models in ablation experiments.

To further increase the difference of activation representations between T–S, we also propose a one-class embedding as the reconstruction block to smooth the abnormal information in low-and high-dimensional representations that cannot be eliminated by AS, here the OCE block for only one $3 \times 3$ convolutional layers^43^ with the stride of 1. Each student’s critical layer corresponds to an AOCE module. Ablation experiments show that the AS module and the OCE block can effectively improve the performance of the model, respectively.

3 $$\begin{aligned} \begin{aligned} M_a^{CL^i} = A(M^{CL^i}) \otimes Conv_{3 \times 3} \end{aligned} \end{aligned}$$

The $A(M^{CL^i})$ represents the feature map output by the student through the attention mechanism module(i.e. AS) to improve the ability of the network to focus on the target area and suppress useless information. The $\otimes$ is convolution operations to filter out these useless information.

Figure 4 displays qualitative comparisons of different AS modules, the student using Sspcab^39^ and CBAM^41^ both fully display the information of structural anomalies in the deep critical layer according to the similarity loss, while the information extracted in the shallow critical layer, the student model using CBAM only extracts information describing the non-anomalous structure of the input data, while the low-level structure information extracted by the student model using Sspcab includes abnormal regions. We also depict the AOCE module in Fig. 2c. AS focuses on anomaly-free activation representations of student outputs (dark blue squares), and OCE improves inter-T–S responses to anomalous regions by smoothing out anomaly information (blue squares).

**Table 1:** Results of anomaly detection.

Category		STPM	SPADE	RIAD	PaDim	CutPaste	DRÆM	MÆIDM	MKD	RD	CSKD	ACSKD
Textures	Carpet	99.8	100	54.6	99.9	89.7	83.4	91.6	68.5	98.7	96.8	**100**
	Grid	88.5	99.8	86.5	95.7	99.0	**100**	**100**	78.9	**100**	**100**	**100**
	Leather	**100**	92.8	98.9	**100**	**100**	98.8	**100**	70.2	**100**	**100**	**100**
	Tile	98.5	47.3	84.6	97.4	98.3	**100**	**100**	95.7	99.8	**100**	**100**
	Wood	98.8	61.5	80.7	98.8	99.2	**99.9**	99.6	94.5	99.0	99.6	99.5
	Average	97.1	80.2	81.1	98.8	98.0	96.4	98.2	81.5	99.3	99.3	**99.9**
Objects	bottle	99.2	95.8	97.1	99.8	99.7	99.4	99.4	99.8	99.9	**100**	**100**
	Cable	99.2	84.8	65.6	92.2	87.9	90.8	94.3	85.2	97.6	99.3	**99.6**
	Capsule	81.8	97.2	72.0	91.5	88.4	92.5	**98.6**	86.0	98.1	97.4	98.4
	Hazelnut	99.5	84.8	81.4	93.3	97.4	99.8	**100**	98.9	**100**	**100**	**100**
	Metal nut	99.4	89.7	62.1	99.2	98.6	99.4	99.5	72.9	**100**	**100**	**100**
	Pill	87.3	88.1	65.7	94.4	90.9	94.9	98.2	89.0	96.5	**98.9**	**98.9**
	Screw	77.4	71.0	59.4	84.4	60.0	98.3	**98.4**	84.4	97.6	95.1	95.2
	Toothbrush	97.5	90.1	98.6	97.2	91.1	99.2	**100**	89.2	99.4	98.6	**100**
	Transistor	97.7	66.7	79.5	97.8	92.2	89.3	94.5	74.2	96.4	98.0	**99.2**
	Zipper	89.7	88.9	98.3	90.9	**100**	98.9	**100**	97.5	98.1	98.4	98.5
	Average	92.9	85.7	78.0	94.4	90.6	96.3	98.2	87.7	98.0	98.6	**99.0**
Total average		93.8	84.3	80.7	95.1	93.0	97.2	98.5	86.8	98.4	98.8	**99.2**

Anomaly detection results on MVTec^17^. The best results of image-level AUROC (%) are highlighted in bold

### Anomaly detection and localization

#### Anomaly localization

In order to detect anomalous samples, each input test is passed to both teacher and student, and learning only the student model without anomalous samples fails in the form of describing out-of-distribution. In contrast, the frozen pre-trained teacher model is able to fully reflect anomalous information in its feature representation. We obtain the anomaly score maps $M^{CL^i}(h,w)$ mentioned calculated by Eq. (1). All anomaly score maps are upsampled $\Phi$ and with Gaussian filtering $g_\sigma$ to reduce their natural noise to form the final anomaly localization map $AL_{map}$.

4 $$\begin{aligned} \begin{aligned} AL_{map} = g_\sigma \sum _{i=1}^I \Phi (M^{CL^i}(h,w)) \end{aligned} \end{aligned}$$

#### Anomaly detection

Since $AL_{map}$ usually does not have an obvious response to the non-abnormal area in the test image, but gives a very high value to the abnormal area, it is reasonable to use the maximum value of the anomaly score map as the image-level evaluation standard $AD_{map}$ for anomaly detection.

5 $$\begin{aligned} \begin{aligned} AD_{map} = max(AL_{map}) \end{aligned} \end{aligned}$$

**Table 2:** Results of anomaly localization.

Category		US	STPM	SPADE	RIAD	PaDim	DRÆM	RD	CSKD	ACSKD
Textures	Carpet	83.8/68.2	98.8/95.3	98.9/–	91.2/65.7	99.0/96.5	90.8/84.7	99.0/97.1	98.6/96.3	**99.3/97.9**
	Grid	79.2/57.6	97.6/90.3	98.3/-	98.2/93.7	96.5/91.0	**99.6/98.5**	99.3/97.4	99.0/96.8	99.2/97.5
	Leather	94.5/94.6	98.9/96.8	99.3/–	99.6/97.5	98.9/97.2	98.9/96.5	99.4/99.1	99.1/98.8	**99.5/99.2**
	Tile	82.0/60.0	92.9/74.0	92.8/-	73.5/42.8	93.9/79.0	**98.7/96.7**	95.6/90.5	96.8/92.8	96.4/93.0
	Wood	85.2/74.4	92.8/86.8	95.3/-	81.0/53.9	94.1/89.2	95.1/83.3	95.3/91.9	95.6/90.9	**96.4/92.8**
	Average	87.3/71.5	96.6/89.0	97.3/-	91.1/76.6	96.5/90.6	96.6/91.9	97.7/95.0	97.8/95.1	**98.2/96.1**
Objects	Bottle	88.7/67.0	97.2/89.8	97.0/–	94.1/82.7	98.2/94.3	98.8/95.6	98.7/96.7	99.2/97.2	**99.2/97.4**
	Cable	76.3/51.9	97.2/91.3	92.3/-	74.6/43.6	96.8/88.1	95.2/81.2	97.2/90.9	97.5/90.9	**98.0/92.5**
	Capsule	93.9/88.9	96.6/78.4	98.4/-	97.7/87.2	**98.6**/92.1	91.8/90.9	**98.6/95.7**	97.8/92.5	98.5/95.4
	Hazelnut	94.1/87.7	97.3/90.3	98.5/–	97.3/90.1	97.9/93.4	**99.6/97.2**	98.9/95.4	99.0/95.8	99.2/96.1
	Metal nut	79.8/57.3	95.3/87.1	97.1/-	84.8/63.5	97.1/91.8	**99.3/97.0**	97.3/92.5	96.7/87.8	97.9/92.5
	Pill	79.8/57.3	95.3/87.1	97.1/-	84.8/63.5	97.1/91.8	**99.7/98.3**	98.2/96.3	98.6/96.8	98.7/96.9
	Screw	92.1/74.5	95.1/80.1	99.1/-	97.6/89.4	98.3/93.4	97.1/92.8	**99.6/98.1**	98.9/95.6	98.8/95.2
	Toothbrush	90.1/73.3	97.4/77.4	98.8/-	98.5/90.1	98.7/91.3	95.3/84.3	99.1/94.3	98.9/93.5	**99.1/93.8**
	Transistor	62.9/40.7	92.5/82.0	88.6/-	77.9/70.0	**97.5/93.3**	89.2/71.8	93.1/79.0	90.1/78.3	95.3/86.5
	Zipper	90.0/71.3	96.7/88.4	98.6/–	98.2/92.8	98.4/94.1	**98.5**/94.2	98.2/**95.6**	97.8/95.0	97.9/94.8
	Average	88.7/68.9	95.9/85.3	96.3/-	91.6/79.8	97.8/92.7	96.5/90.3	97.9/93.3	97.5/92.3	**98.3/94.1**
Total average		85.3/69.5	95.9/86.4	96.5/–	90.6/76.7	97.3/91.9	96.5/90.8	97.8/93.8	97.5/93.2	**98.2/94.7**

Anomaly Localization results on MVTec^17^. the TOP results for the pixel-level AUROC and PRO are highlighted in bold

## Experiment

In this section, the unsupervised anomaly detection and localization capabilities of CSKD and ACSKD (CSKD with AOCE module) are extensively evaluated and compared with recent SOTA methods. In addition, the impact of the various components of the proposed method on the final result is evaluated through ablation studies on the public benchmark MVTec dataset^17^. Finally, the superiority of the proposed method is demonstrated by comparing ACSKD with state-of-the-art unsupervised detection methods on the DAGM dataset^19^ and one-class anomaly detection benchmark datasets.

### Anomaly detection and localization

#### Dataset

The benchmark of MVTec^17^ contains 15 categories of objects and textures with a total of 3629 images for training and 1725 images for testing. The training set only includes non-anomalous images. All images have a resolution between $700 \times 700$ and $1400 \times 1400$ pixels

**Experimental setting.** All images in MVTec^17^ are resized to a uniform resolution of $256 \times 256$. We follow previous work to apply Wide-Resnet-50 as the backbone of teacher and student encoders. We use Adam^44^ optimizer with $\beta = (0.5, 0.999)$. The learning rate is set to 0.005. We train 200 epochs with a batch size of 16. A Gaussian filter with $\sigma = 4$ is used to smooth the anomaly score map. The query is performed every 10 epochs and save the model, we select the model with the relatively optimal hard-coded number of epochs according to the results of the query.

**Evaluation criterion.** The widely used area under the receiver operating characteristic curve (ROCAUC)^21,36^ is used as the evaluation metric for detection and localization. At the same time, in order to prevent AUROC from being biased toward large abnormal areas, the per-region-overlap curve (PROAUC)^36^ that can treat all abnormal regions equally is also considered as the evaluation standard for abnormal localization. the false positive rate in PROAUC is lower than 0.3. For ROCAUC or PROAUC criterion, higher values mean that the model performs better for anomaly detection and localization. These evaluation metrics are all used for querying and testing.

**Figure 5:**
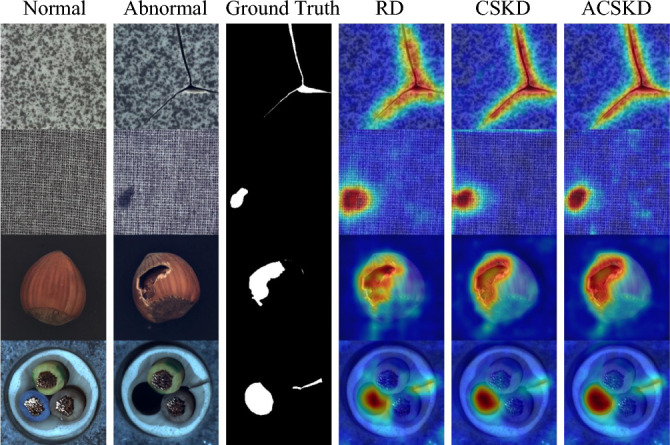
The normal samples as a reference are shown in the first column. The last two column show the anomaly maps generated by our implementation of Reverse Distillation^18^, CSKD, and ACSKD, respectively. The last column shows the direct anomaly map output of ACSKD.

**Figure 6:**
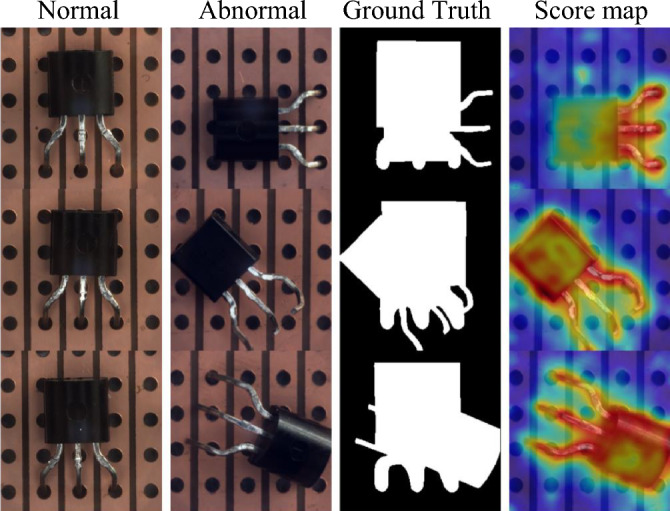
ACSKD produces an anomaly map that correctly localizes the misplace regions of transistor. But the discrepancy with the ground truth marks the area where cover both misplaced and original areas that increases the performance error.

**Figure 7:**
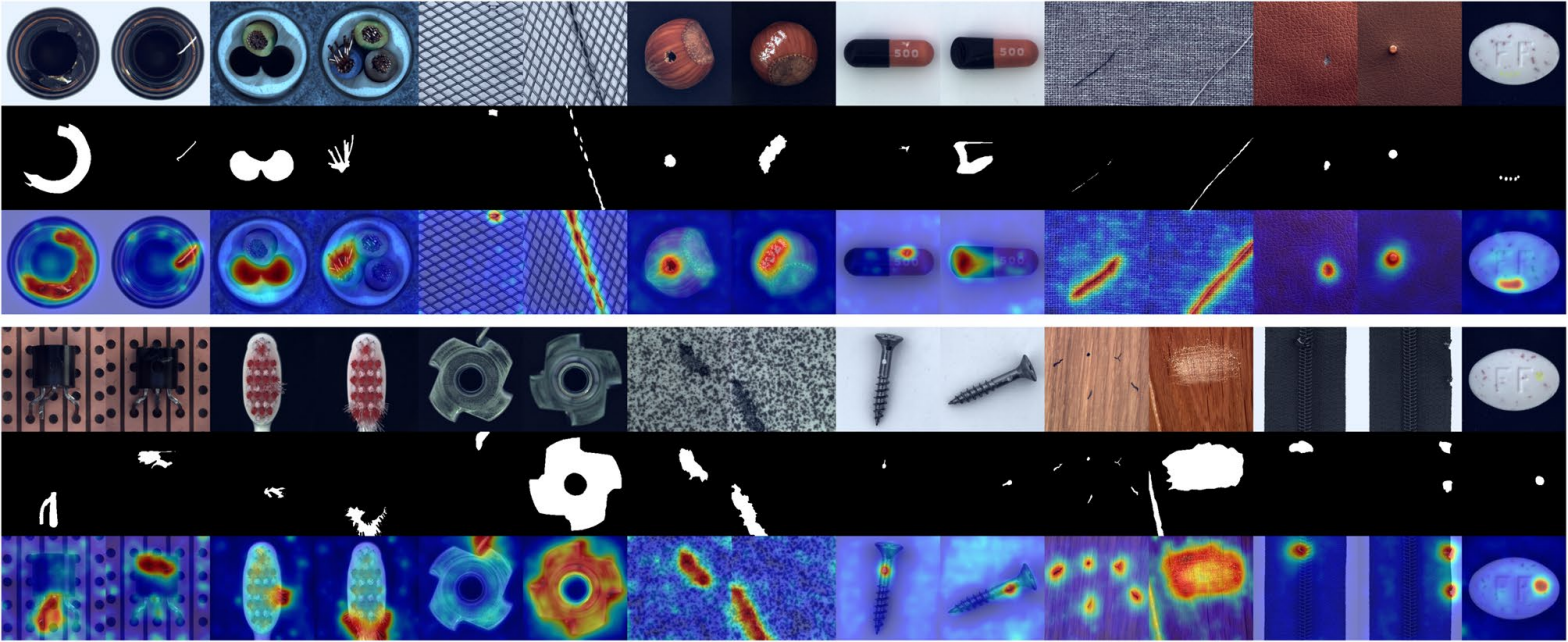
Qualitative samples of all categories in MVTec AD^17^ achieved by ACSKD are shown.

**Figure 8:**
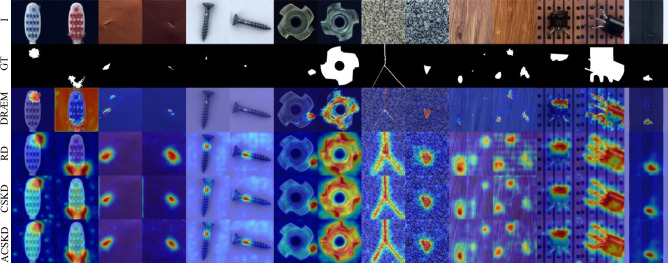
Qualitative comparison of CSKD and ACSKD to the anomaly detection methods DRÆM^26^ and RD^18^ on the MVTec dataset^17^. The original image (I), the ground truth map (GT), and the anomaly map for four methods are shown.

## Experimental results and discussions

### For anomaly detection

Table 1 quantitatively compares ACSKD with STPM^35^, SAPDE^8^, RIAD^45^, PaDim^11^, CutPaste^32^, DRÆM^26^, MÆIDM^12^, MKD^5^ and RD^18^ on the image-level surface anomaly detection task. CSKD significantly outperforms all recent anomaly detection methods, achieving the highest image-level AUROC in 13 out of 15 categories and achieving considerable accuracy in the remaining two categories, respectively reaching second and third among all methods. For textures and objects, CSKD and ACSKD achieve new optimal metrics with AUROC of 99.3%/99.9% and 98.8%/99.0%, respectively.

### For anomaly localization

A recent comparison of state-of-the-art methods on pixel-level anomaly localization are summarized in Table 2. These methods include Uniformed Student (US)^36^, STPM^35^, SAPDE^8^, RIAD^45^, PaDim^11^, DRÆM^26^ and RD^18^. ACSKD produced competitive results with the previous best-performing method with an average AUROC score of 98.2% and an AUPRO inhibition of 94.7% all the state-of-the-art. The quality comparison of CSKD and ACSKD with the optimal method reverse distillation is shown in Fig. 5. The proposed method achieves a significant improvement in anomaly segmentation accuracy.

Detailed inspection shows that some detection errors can be attributed to inaccurate ground-truth label annotations for some of the anomalies in the MVTec dataset^17^. As shown in Fig. 6, ACSKD correctly locates the missing regions in the transformer and gives an anomaly score map. However, the ground truth annotates both the missing and initial regions, which increases the possibility of errors in detection accuracy. These ambiguous annotations also affect the accuracy of the pixel-level AUROC and PROAUROC values of the evaluation method. We present more examples of anomaly detection and localization in Fig. 7 and the qualitative comparison of CSKD and ACSKD to the recent RD^18^ and DRÆM^26^ methods is shown in Fig. 8.

### Surface anomaly detection and one-class anomaly detection

To further evaluate the generality of the proposed method, we perform surface anomaly detection on the DAGM^19^ benchmark and experiments on three benchmarks commonly used for one-class anomaly detection: MINST^46^, F-MINST^47^, and CIFAR-10^48^.

The DAGM dataset contains 10 classes of textured objects with small anomalies that are visually very similar to the background. This dataset is often used as a benchmark for methods such as supervised or semi-supervised surface anomaly detection, which makes this dataset useful for unsupervised methods. MNIST: 60k grayscale images of handwritten digits 0-9 for training and 10k for testing. F-MNIST: The number and form are the same as MINST, the difference is that the dataset categories. The resolution of the pictures in both is $28 \times 28$. CIFAR-10: 50K training and 10K test images with $32 \times 32$ in 10 categories.

For the DAGM dataset^19^, we used a new version processed in previous work^12^ that is more suitable for unsupervised surface anomaly detection as the benchmark. PaDim^11^, DRÆM^26^ and RD^18^, which achieved superior performance on the DAGM dataset^19^, were selected as the baselines for surface anomaly detection. The baselines in one-class anomaly detection are LSA^49^, HRN^50^, OCGAN^51^, DASVDD^52^ and RD^18^.

**Table 3:** Results of surface anomaly detection .

Methods	US	STPM	PaDim	DRÆM	CutPaste	MKD	RD	CSKD	ACSKD
Image-level AUROC	–	87.1	94.1	88.4	90.6	76.7	90.4	95.2	**97.2**
Pixel-level AUROC	67.4	93.6	96.1	79.8	–	–	94.7	97.2	**97.6**
PRO	41.4	81.7	–	88.8	–	–	89.4	93.7	**94.8**

The top results of image/pixel-level AUROC(%) on DAGM dataset^19^ are marked in bold

**Figure 9:**
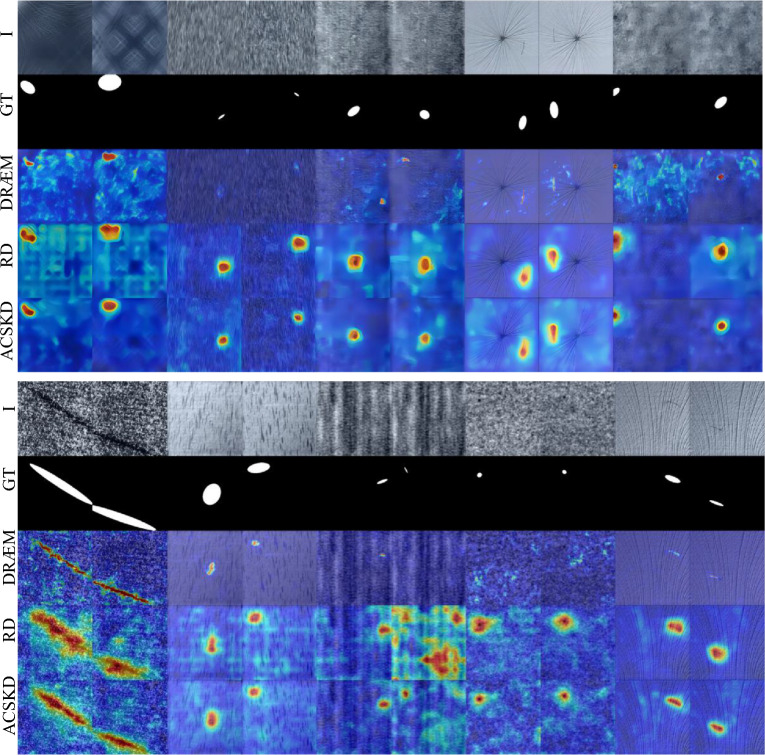
Qualitative examples for the DAGM dataset^19^. The original image Input, the ground truth map GT, the anomaly map produced by DRÆM^26^ and RD^18^, and the ACSKD anomaly map are shown.

Tables 3 and 4 summarize the quantitative results of these two benchmarks. Remarkably, our approach produces excellent results. A comparison of the anomaly score maps generated by RD^18^ and DRÆM^26^ with ACSKD is shown in Fig. 9.

**Table 4:** Results of AUROC on one-class anomaly detection.

Methods	LSA	HRN	OCGAN	DASVDD	RD	ACSKD
MINST	97.4	97.6	97.7	97.7	96.9	**99.4**
F-MINST	–	92.8	92.4	92.6	93.6	**95.6**
CIFAR-10	64.0	71.3	65.7	66.5	85.0	**86.1**

The methods with top AUROC(%) on one-class anomaly detection are marked in bold

### Ablation analysis

All ablation experiments use the mean of all classes in the MVTec dataset as the comparison parameter.

**Table 5:** Ablation study on assistant student and one-class embedding block.

Metric	Pre	Pre+OCE	Pre+AS	Pre+AS+OCE
Image-level AUROC	98.8	98.6	99.0	**99.2**
Pixel-level AUROC	97.5	97.8	97.7	**98.2**
PRO	93.2	93.6	93.6	**94.7**

Significant values are in bold.

We study the effect of assistant student block (AS) and one-class embedding (OCE) block in the proposed AOCE module and report the numerical results in Table 5. We take CSKD (Pre) without the AOCE module as the baseline. The AS improves the representation capabilities of student models, and the OCE block effectively weakens the vanishing representations discrepancy between teacher and student on abnormal areas by smoothing the abnormal information contained in the low- and high-dimensional feature representations extracted by the student model with the assistant student. Experiments show that the proposed AOCE has the ability to achieve more accurate anomaly detection and localization.

**Table 6:** Ablation study on different backbones.

Backbone	R18	R34	R50	WR50
Image-level AUROC	97.4	98.6	97.9	**98.8**
Pixel-level AUROC	96.9	97.1	97.0	**97.5**
PRO	91.8	92.0	92.2	**93.2**

Significant values are in bold.

Individual components of the proposed model CSKD without the AOCE module are evaluated by subsequent experiments. We explore the different backbone networks as the teacher and student models in Table 6. Intuitively, a deeper and wider backbone can obtain more complex feature representation information, thereby achieving more accurate anomaly localization. Of course, a deeper network will also cause the similarity of T–S responses to anomalous areas.

**Table 7:** Ablation study on single or multi-scale critical layers.

Layers	I/P-AUROC	PRO	Layers	I/P-AUROC	PRO
$L^1$	90.9/93.7	88.3	$L^{23}$	98.6/97.4	92.2
$L^2$	96.9/96.2	91.9	$L^{234}$	96.5/95.2	85.7
$L^3$	95.5/95.7	86.1	$L^{1234}$	95.7/96.5	89.9
$L^4$	85.1/84.5	50.0	$L^{123}$ (Ours)	**98.8**/**97.5**	**93.2**
$L^{12}$	96.1/96.4	92.4			

Significant values are in bold.

Table 7 shows the impact of different network layers on the performance of anomaly detection. $L^{23}$ is close to the multi-scale combination used in this paper as it contains both local texture information and global structure information.

**Table 8:** Quantitative comparison with different query epochs.

Query	20	40	50	100	200	10(Ours)
Image-level AUROC	98.0	97.4	97.4	97.0	95.3	**98.8**
Pixel-level AUROC	97.5	97.2	97.6	96.9	96.0	**97.5**
PRO	93.0	92.3	93.0	85.2	88.7	**93.2**

Significant values are in bold.

We investigate the effect of different interrogation intervals on the performance of the model and report the results in Table 8. A query interval of 10 epochs can get the model hard-coded epochs relatively accurately.

**Table 9:** Performance of different AS modules.

AS	Sspcab	CBAM	SeNet	EcaNet
Image-level AUROC	97.8	98.3	98.7	98.8
Pixel-level AUROC	98.1	97.8	97.8	98.0
PRO	94.5	94.0	93.9	93.9

Finally, we show the average detection performance of different AS modules across all categories in the MVtec dataset and report in Table 9. We can see that the AS module composed of Sspcab outperforms others in anomaly localization, while EcaNet is superior in the generalization of detection and localization of all categories.

### Discussion

We observe that the proposed method still has certain limitations. For anomaly localization, there is still a certain gap in the accuracy of defect edge segmentation compared with methods based on image reconstruction (DRÆM^26^, etc.), because the two methods complete anomaly detection from the image-level and feature-level respectively. and positioning. The method based on knowledge distillation, the T–S model cannot complete the segmentation of abnormal areas through pixel-level representation in the low- and high-dimensional feature subspace. Secondly, compared with previous work, the proposed method uses the AOCE module to alleviate the impact of noise on the model during testing to a certain extent. However, how to prevent the student model from extracting abnormal information or using the assistant module to completely eliminate this information is still the next research work. Finally, how to solve the universality of AOCE modules for all industrial categories is also a key issue. For some industrial product categories, the performance of different AS modules shows great differences. Therefore, how to explain this phenomenon in order to propose an AOCE with better versatility becomes the focus of the next work.

For the future research, RD^18^ and DeSTSeg^27^ combined knowledge distillation and image reconstruction and achieved excellent performance in the field of surface defect detection. The performance of the Sspcab module in some industrial images also proved the importance of defect reconstruction. Therefore, the next step of research will focus on better combining image reconstruction and knowledge distillation to achieve a new paradigm for surface defect detection of industrial products.

## Conclusion

We proposed a new knowledge distillation paradigm, *Cosine Similarity Knowledge Distillation*, for anomaly detection. The proposed method effectively addresses the data manifold between the same T–S models, which results in the disappearance of feature representations and further improves the traditional knowledge distillation to anomaly detection accuracy. Additionally, we introduced an assistant student and OCE block to build the attention one-class embedding module to further act as an assistant module while increasing the variability of the model’s response to anomalous regions. Extensive experiments show that our method significantly outperforms previous state-of-the-art unsupervised methods for anomaly detection and localization including surface anomaly detection and novelty detection.

## Data Availability

The datasets used and/or analysed during the current study available from the corresponding author on reasonable request.
